# On the Poison of Venomous Snakes and the Methods of Preventing Death from Their Bite

**Published:** 1909-12

**Authors:** 


**On the Poison of Venomous Snakes and the Methods of Preventing Death from their Bite.** Reprinted papers by the late Sir JOSEPH FAYRER, Bt., K.C.S.I., M.D., Sir LAUDER BRUNTON, Bt., LL.D., M.D., and Major LEONARD ROGERS, I.M.S., M.S. Pp. 174. London: Macmillan & Co. 1909.

This is a reprint of papers which appeared in the *Proceedings of the Royal Society* between the years 1873 and 1904. It is a record of careful research and of conclusions based on two hundred experiments upon animals. The greater part of the book deals with the physiological action of venoms, proving the practically identical character of the poison of both colubrine and viperine snakes, though the local action of the latter is shown to be more pronounced. This corresponds with clinical experience, the bite of a viper causing intense pain, with extravasation and swelling, going on to sloughing when life is sufficiently prolonged ; whereas in many cases of cobra bite there is but little to see locally except the double puncture. The rapid putrefaction developing in venom poisoning, referred to by the authors, has since been shown by Ewing and Martin to be due to the fact that the venom destroys the bactericidal power of the serum.

The book closes with the experimental proof of the value of the permanganate treatment of snake bite. Animals which had received three times the minimum lethal dose of venom survived, though an interval of ten minutes was in some cases allowed to elapse before the treatment was applied, whilst a "control" died in four and a half hours. If Brunton's simple permanganate apparatus were in the hands of village headmen and the minor officials that abound in Indian villages, not a few of the 20,000 lives lost annually in that country from snake bite might be saved. The researches of Frazer and Calmette have given us the more perfect and potent treatment by antivenene, but its cost and method of application limit it to the hands of the few, whereas Brunton's lancet and permanganate might be scattered broadcast.

